# Proof-of-Concept Study: a Mobile Application to Derive Clinical Outcome Measures from Expression and Speech for Mental Health Status Evaluation

**DOI:** 10.1007/s10916-020-01671-x

**Published:** 2020-11-11

**Authors:** Francesco Luke Siena, Michael Vernon, Paul Watts, Bill Byrom, David Crundall, Philip Breedon

**Affiliations:** 1 Nottingham Trent University, Nottingham, UK; 2 Signant Health, London, UK

**Keywords:** Facial expression, Clinical outcome assessments, Video analysis, Mental health

## Abstract

This proof-of-concept study aimed to assess the ability of a mobile application and cloud analytics software solution to extract facial expression information from participant selfie videos. This is one component of a solution aimed at extracting possible health outcome measures based on expression, voice acoustics and speech sentiment from video diary data provided by patients. Forty healthy volunteers viewed 21 validated images from the International Affective Picture System database through a mobile app which simultaneously captured video footage of their face using the selfie camera. Images were intended to be associated with the following emotional responses: anger, disgust, sadness, contempt, fear, surprise and happiness. Both valence and arousal scores estimated from the video footage associated with each image were adequate predictors of the IAPS image scores (*p* < 0.001 and *p* = 0.04 respectively). 12.2% of images were categorised as containing a positive expression response in line with the target expression; with happiness and sadness responses providing the greatest frequency of responders: 41.0% and 21.4% respectively. 71.2% of images were associated with no change in expression. This proof-of-concept study provides early encouraging findings that changes in facial expression can be detected when they exist. Combined with voice acoustical measures and speech sentiment analysis, this may lead to novel measures of health status in patients using a video diary in indications including depression, schizophrenia, autism spectrum disorder and PTSD amongst other conditions.

**Supplementary Information:**

The online version contains supplementary material available at 10.1007/s10916-020-01671-x.

## Introduction

Mental health disorders are among the most common health conditions affecting the adult population [1]. Anxiety and depressive disorders are estimated to affect over 322 million and 264 million people worldwide respectively [2]. The Global Burden of Disease Study 2017 estimated that depressive disorders were the third highest cause of years lived with disability (YLD) globally [3]. Over 50% of the general population in middle- and high-income countries are estimated to suffer from at least one mental health disorder in their lifetimes [4] .

In the EU, it is estimated that each year almost 40% of the population suffer from some form of mental health condition [5], with anxiety disorders affecting around 14% of the population, and insomnia and major depressive disorders the next highest affecting 7% and 6.9% respectively [5]. In England the prevalence of mental health disorders is predicted to rise by 14.2% over the 20-year period from 2007 to 2026 [6]. Spending in health and social care related to mental health in England is estimated to account for around 12% of the National Health Service budget and cost approximately US$28 billion a year [6].

With high and increasing global prevalence of mental health disorders, there is a need to evaluate the progress of patients in effective and efficient ways both in routine care and to assess intervention effects in clinical research.

Speech patterns, and their changes over time, provide potential insights into the health status of patients. For example, a study of patients with Parkinson’s disease showed that around 75% of patients exhibit some form of vocal impairment [7]; and voice acoustical changes can be good predictors of early onset of the disease [8]. In patients with depression, certain aspects of speech, such as speaking rate and pitch variability, have been shown to correlate well with conventional measures of the severity of depression such as the Hamilton Depression Rating Scale [9].

Smartphone technology has simplified the collection of digital voice samples. For example, phonation tests for Parkinson’s disease patients have been developed in clinical research mobile applications (apps) using both Apple Research Kit [10] and on the Android platform [11]. This opens the possibility of using such inexpensive techniques in large-scale clinical trials and within routine care.

More recently, machine learning techniques afford greater opportunity to derive insights from complex data such as voice samples. One study, for example, used machine learning techniques to create a model based on an initial input of 370 extracted linguistic features that was able to adequately distinguish between Alzheimer’s disease patients and healthy controls based on analysis of short narrative samples elicited with a picture description task [12].

Similarly, video analysis may provide valuable health status insights. Depression is often associated with a flattening of positive emotional responses [13] which can be expressed in blunted expression. Promising work has been conducted on the extraction of facial expression based on computer recognition of the relative position of facial landmarks – for example in aiding the diagnosis of autism spectrum disorders in young children while watching short video content designed to elicit certain emotional responses such as surprise and happiness [14].

Objective measures based on expression and speech indices derived from video diary data may provide additional insights into the health status of patients suffering from certain mental health conditions such as depression, anxiety, schizophrenia, autism spectrum disorder, PTSD and others. Current measurement approaches in clinical trials typically centre around subjective clinical outcome assessments (COAs) made by the patient individually or by a clinician during a psychiatric interview. The same measures are also used to measure disease severity in clinical practice, for example UK primary care physicians are funded through the Health Service’s Quality and Outcomes Framework (QOF) for assessing the severity of symptoms of depression by using one or a number of self- and clinician-assessed COAs [15]. In some cases, the subjectivity of these measures may make them less sensitive to detecting intervention effects. In an analysis of new drug applications approved by the FDA between 1985 and 1997, for example, Khan et al. [16] reported that over 50% of adequate-dose new antidepressant treatment arms failed to demonstrate statistically significant separation from placebo.

### Video diary Mobile application (VDMA): An expression- and speech-tracking mobile platform

**Fig. 1:**
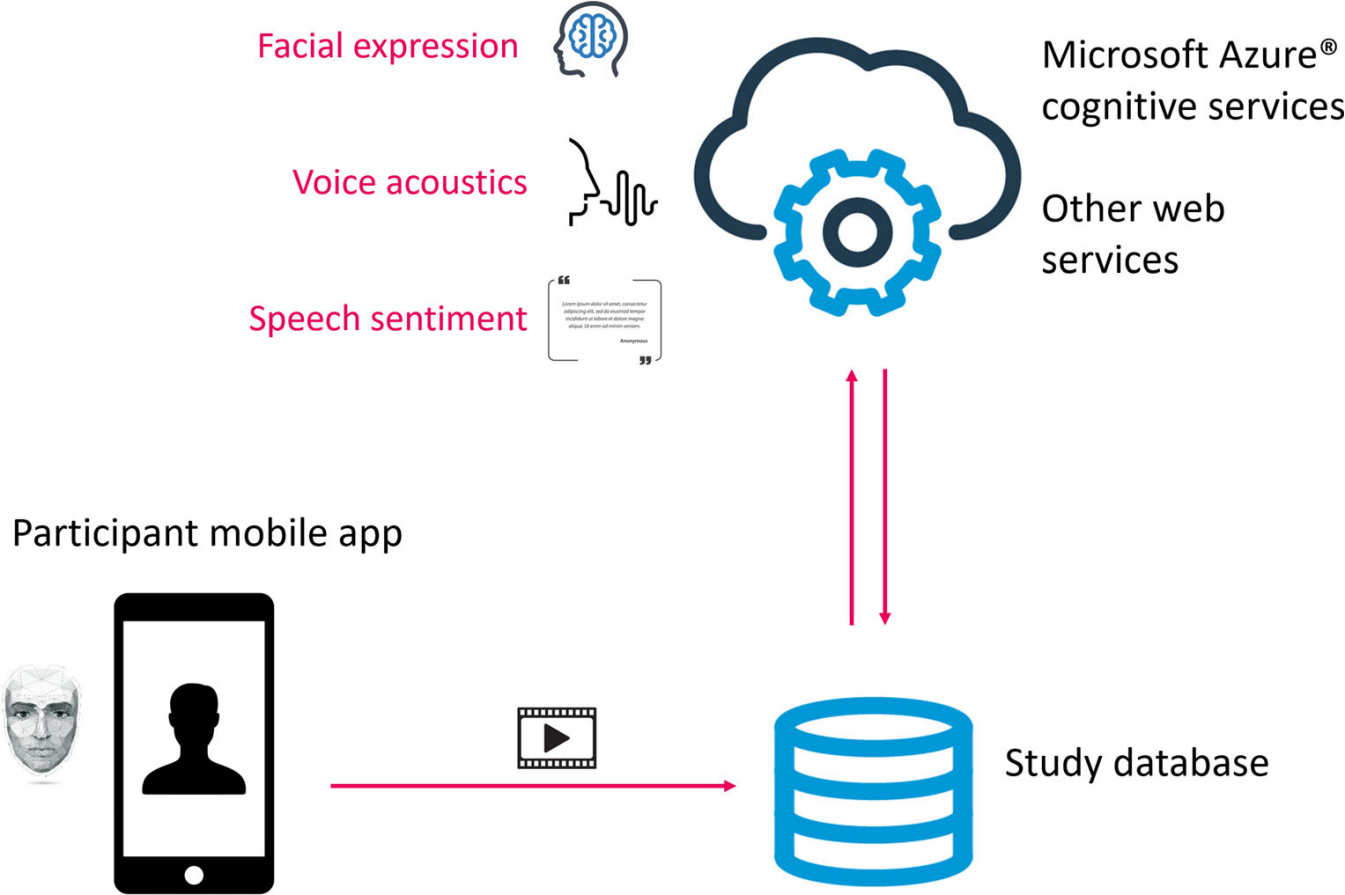
The VDMA and associated cloud analytics software solution

It was our intention to create a method of assessing mental health status that removed subjectivity from the measurement process and may provide a valuable adjunct or alternative to current subjective assessments. To this end we developed a mobile application and cloud analytics software solution to extract possible health outcome measures based on expression, voice acoustics and speech sentiment analysis from video diary footage collected using an Android smartphone app (Fig. 1). The app is intended to be used by patients to record how they feel in their own words longitudinally before, during and after a treatment intervention. In depression, for example, patients may be prompted to describe through short videos how they are feeling emotionally and physically, and how these feelings impact their daily life. In addition to providing a good reference to enable self-assessment of change over time, the analysis of resulting video in terms of expression, voice acoustics and speech sentiment may lead to composite endpoints that provide a useful objective measure of health status and disease severity to enable longitudinal changes to be tracked and the effects of interventions measured.

For the expression assessment component of the VDMA, self-recorded video segments collected using the app were stored and facial expression metrics were extracted for each video frame using Microsoft Azure® Cognitive Services (Microsoft Corporation, Redmond, WA, USA). This provided a frame-by-frame measure of the relative presence of eight expressions: anger, contempt, disgust, fear, happiness, sadness, surprise and neutral. Frame data was combined to generate an expression profile which could be inspected and summary measures extracted. This proof-of-concept study aimed to obtain preliminary data to assess the validity of this expression detection component of the VDMA system.

## Methods

The first step in demonstrating a proof-of-concept is to confirm that the system can accurately identify emotions via a video recording of participants’ faces. To do this, we need a ground truth of what participants are feeling at a particular point in time. While it is possible to ask participants what emotion they were feeling (which we did), there is no guarantee that self-report is accurate due to lack of introspection and reporting biases (one of the criticisms of self-evaluations). Instead we opted to verify the efficacy of the facial encoding by presenting participants with images from the International Affective Picture System (IAPS) [17], which are known to evoke specific emotions in viewers.

To facilitate this, we modified the VDMA to present images on screen that were intended to evoke an emotional response, and simultaneously capture associated facial expression using video footage recorded using the selfie camera during image presentation. Healthy volunteers aged between 18 and 65 years, non-pregnant and without heart condition or any condition or ongoing treatment affecting emotional state, were requested to view 21 validated images from the IAPS database [17] (Online Resource 1) with simultaneous capture of video footage for 5 s following image presentation. Images were selected to generate the following emotional responses: anger, disgust, sadness, contempt, fear, surprise and happiness (3 images for each emotional response). A reset period of 4 s was applied between each image presented in which a neutral image was displayed for 3 s, followed by a fixation cross in the centre of the screen for a duration of 1 s in order to cue the participant to focus their attention in the centre of the screen. After each image was presented, participants were also asked to provide self-ratings of their emotional response associated with each image using a 10-point numeric response scale to rate each possible emotion (0 = none, 9 = extremely). The combined reset period and time taken for self-rating of emotional response was considered more than adequate to avoid carryover of responses between images. Expression analysis was performed using Microsoft Azure Cognitive Services which provided a measure of the estimated relative influence of each emotion within each frame of the captured video segment, and the association with IAPS data was assessed for each image quantitatively and qualitatively.

Eligible participants based in the UK were invited to participate in this single centre, single visit study. Participants provided written informed consent to participate. The study was approved by the Nottingham Trent University Independent Ethics Committee, UK.

Emotional response profiles were also summarised into two measures: valence (unpleasant to pleasant) and arousal (calm to exciting) ranging from 0 to 9 on a frame-by-frame basis. Each frame was assigned a dominant emotion determined as the emotion identified to increase the most from the previous video frame, and this frame was assigned valence and arousal scores associated with the dominant emotion based on the relationships estimated from Lang [18] (Table 1). Overall valence and arousal scores were calculated by averaging the per-frame values. This method was selected with the aim of limiting the influence of resting expressions on the estimates of valence and arousal responses to the IAPS images.

**Table 1:** Valance and arousal scores assigned to per-frame dominant emotions extracted from video footage recorded by the VDMA, based on [18]

Emotion	Valance	Arousal
Anger	2	8
Contempt	2	8
Disgust	1	8
Fear	3	7
Happiness	8	3
Sadness	1.5	4.5
Surprise	5	8
Neutral	5	0

Data were analysed quantitatively and qualitatively. In the quantitative analysis valence and arousal scores were assessed in their ability to predict the IAPS image valence and arousal scores using linear mixed-effects multi-level models, controlling for random effects using random by-participants and by-stimulus image intercepts. In the qualitative analysis, each 5 s expression profile was categorised manually using the following categories: a positive response in line with the target expression, a non-response, or a false-positive response (i.e., an emotional response was detected, but a different response to that intended by the specific IAPS image).

The validity of the IAPS images in evoking the intended emotion in the study sample was evaluated with reference to the self-assessment scores received for each image.

## Results

Forty participants (ages 21–57 years; Sex: M (20), F (19), Not stated (1)) participated in this proof-of-concept study (Table 2). One participant was excluded from the analysis as the video data were unusable due to placing their hands over their face during most video segments.

**Table 2:** Participant characteristics

Variable	*N* **= 40**
Age (years)	
Range	21–57
Mean [SD]	30.9 [10.3]
Gender	
Female	19
Male	20
Not stated	1

### Quantitative analysis of valence and arousal

The ordering of mean valence scores estimated by the VDMA was broadly in line with those provided with the IAPs images (Table 3). This relationship was less apparent for the arousal estimates (Table 3). A significant main effect of VMDA valence scores was found when comparing the intercept only model (Akaike information criterion (AIC) = 22,298), containing only intercepts and random effects to predict the IAPS valence scores, and an additive model in which VMDA score was added as a fixed effect (AIC = 22,278, χ^2^ (1) = 21.93, *p* < .001). This significant main effect provides some evidence that the valence scores estimated by the VMDA predict the mean valence scores associated with the IAPS images. Similarly, a comparison of the intercept only model (AIC = 14,554), containing only intercepts and random effects to predict the IAPS arousal scores, and an additive model containing the VDMA arousal scores as a fixed effect (AIC = 14,552), found a significant main effect for VDMA arousal scores (χ^2^ (1) = 4.11, *p* = .04). This also provides some support that the VDMA arousal scores predict the mean arousal scores associated with the IAPS images.

**Table 3:** IAPS valence and arousal measures and corresponding values estimated from the VDMA

Emotion	Valence (mean [SD])		Arousal (mean [SD])	
	IAPS	VDMA	IAPS	VDMA
Anger	1.85 [1.28]	3.71 [2.06]	6.89 [2.18]	5.13 [1.30]
Disgust	2.14 [1.38]	3.76 [2.20]	6.54 [2.21]	4.92 [1.18]
Sadness	2.26 [1.50]	3.84 [1.95]	4.37 [2.09]	4.95 [0.97]
Contempt	2.30 [1.76]	3.78 [1.99]	6.21 [2.36]	4.91 [0.93]
Fear	3.63 [1.86]	3.84 [2.08]	6.69 [1.88]	4.85 [0.95]
Surprise	6.67 [1.82]	4.32 [2.13]	6.26 [2.11]	4.74 [0.96]
Happiness	7.71 [1.43]	5.55 [2.40]	4.45 [2.24]	4.25 [1.29]

### Qualitative analysis of response type

**Fig. 2:**
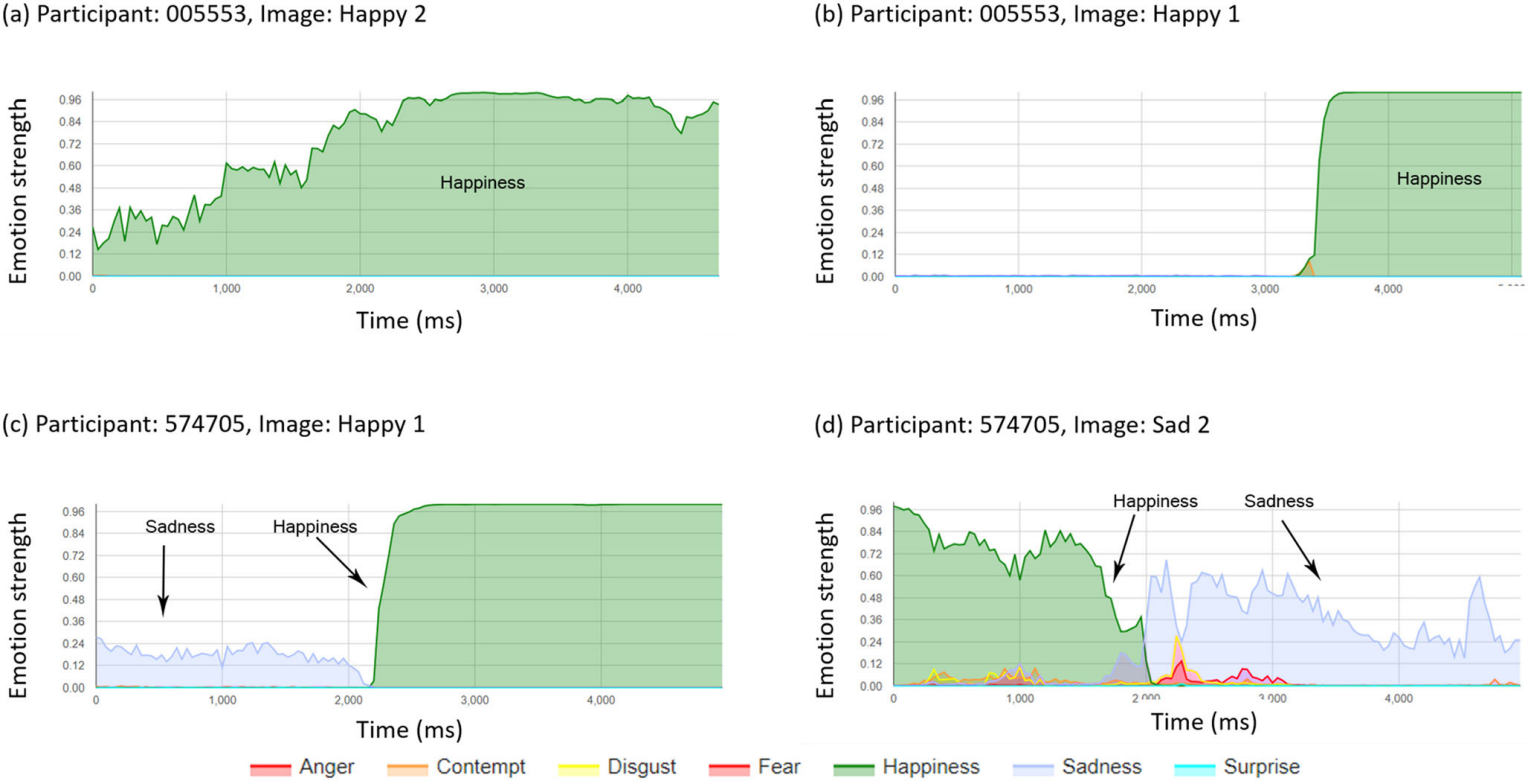
Representative positive responder profiles

**Fig. 3:**
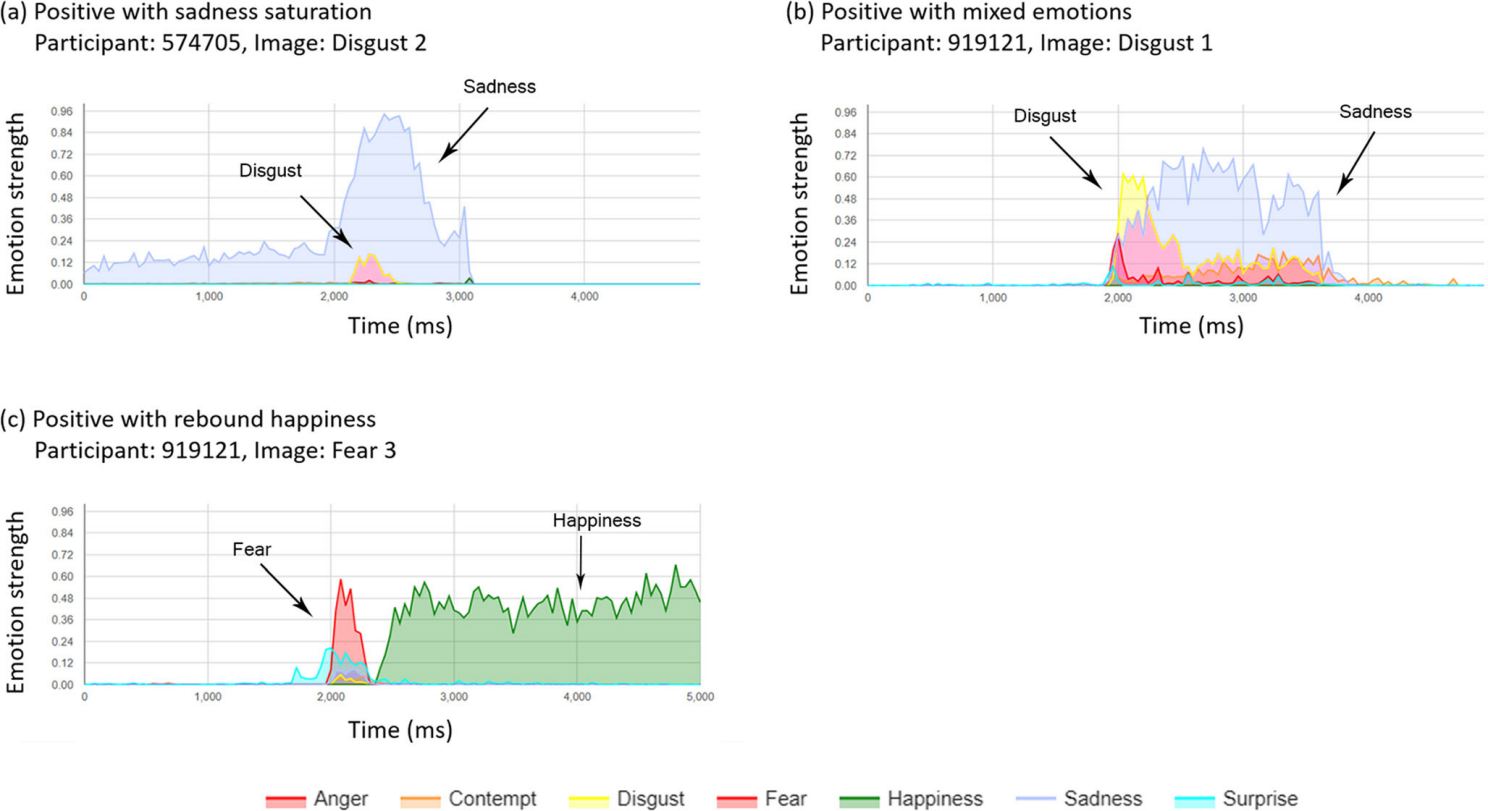
Representative positive responder profiles with mixed profiles

**Fig. 4:**
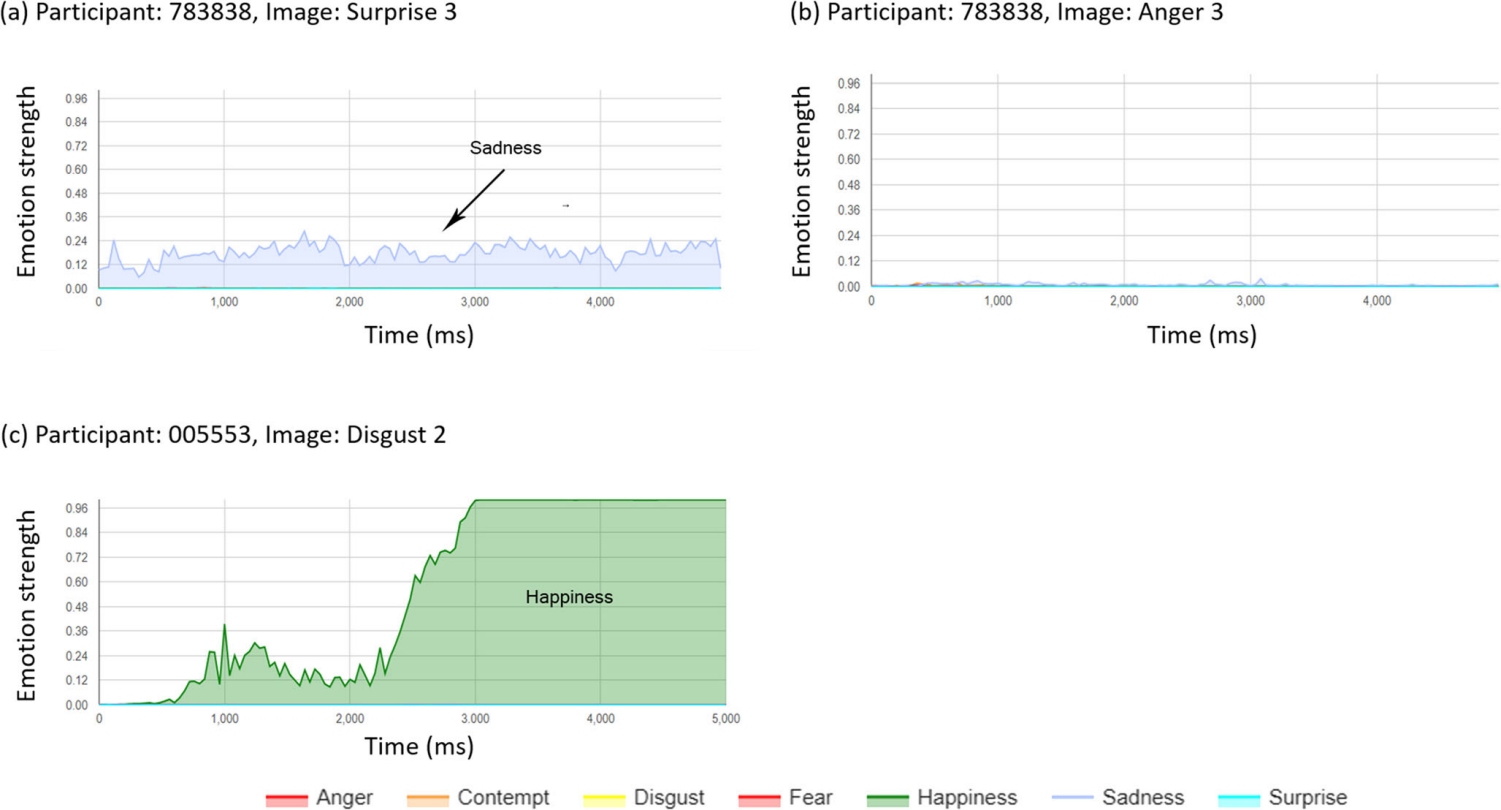
Representative non-responder (**a**, **b**) and false positive (**c**) profiles

The expression data were hard to summarise via automated summary measures because (i) some participants exhibited a resting face expression that was not neutral, and this could mask the detection of the target expression, (ii) some participants showed the target expression response, but this was superimposed by one or more other dominant expressions, and (iii) some target expression profiles may be dominated by a later rebound expression (e.g. happiness following a fear response). For these reasons, it was determined to categorise individual image response profiles manually by inspection by a single reviewer. One additional participant was excluded from this analysis as they provided only 8/21 video segments. Each image profile (*n* = 819 profiles from 39 participants, see Figs. 2-4) was reviewed qualitatively to assess whether the target expression could be observed via a peak relative to the starting expression. Types of response and non-response to each image was classified into a number of observed patterns:

- Positive responder, where a clear response of the target expression was dominant for part of the response profile (Positive-responders, Fig. 2).
- Positive responses with interference from other expression profiles, including responses saturated by another dominant expression (Positive-saturated, Fig. 3a), responses mixed with a number of other expressions of similar magnitude (Positive-mixed, Fig. 3b) and responses that also show a rebound of a further (non-target) emotion (Positive-rebound, Fig. 3c).
- False-positive, an expression detected other than the target expression.
- Non-responder, where a neutral expression, or no change in expression was observed (Fig. 4).

#### Responses in agreement with the target response

100 profiles (12.2%) were categorised as containing a positive response in expression in line with the target expression (Table 4). Of these, target images expected to elicit responses of happiness and sadness provided the highest frequency of responders: 48 and 25 positive responses representing success rates of 41.0% and 21.4% respectively. Relatively few positive responses were observed in response to images targeting expressions of anger, contempt, disgust, fear and surprise.

**Table 4:** Proportions of responders and non-responders from categorisation of expression response profile by IAPS image type

Expression Category	Target Emotion (n)							
	Anger	Contempt	Disgust	Fear	Happiness	Sadness	Surprise	TOTAL
Responder	6	8	8	3	48	25	2	100
Positive responder	3 (50%)	5 (62.5%)	–	–	44 (91.7%)	22 (88%)	–	74 (74%)
Positive-saturated	1 (16.7%)	2 (25%)	5 (62.5%)	–	2 (4.2%)	–	1 (50%)	11 (11%)
Positive-mixed	2 (33.3%)	1 (12.5%)	3 (37.5%)	2 (66.7%)	2 (4.2%)	2 (12%)	–	12 (12%)
Positive-rebound	–	–	–	1 (33.3%)	–	–	1 (50%)	2 (2%)
Non-responder	111	109	109	114	69	92	115	719
False positive	25 (22.5%)	24 (22%)	33 (30.3%)	22 (19.3%)	4 (5.8%)	7 (7.6%)	21 (18.3%)	136 (18.9%)
Non-responder	86 (77.5%)	85 (78%)	76 (69.7%)	92 (80.7%)	65 (94.2%)	85 (92.4%)	94 (81.7%)	583 (81.1%)

#### Non-responders and false positive responses

719 profiles (87.8%) were categorised as containing a non-response relative to the target image, with 18.3% to 30.3% of these showing false positive responses in favour of a non-target expression for all expressions except happiness and sadness (Table 4). For these emotions, few false-positives were observed amongst the non-responders: 4 (5.8%) and 7 (7.6%) for happiness and sadness respectively.

#### Detection of any change in facial expression

We further categorised each participant in terms of their ability to present a change of expression in response to the images presented. 9/39 (23%) we identified as responders, having at least 5/21 responses in line with the target expression of the images presented (Table 5). Of these, 7 participants we categorised as expressive responders, those that provided a high number of false positives in addition to responses in line with the target expression. Amongst the non-responders, 29 (74.4%) of participants provided 0–3 positive responses to the target expression.

**Table 5:** Distribution of overall responder types of the participants (*n *= 39)

Responder type	N = 39
Responder	2 (5.1%)
Responder (expressive)	7 (17.9%)
Non-responder	29 (74.4%)
Non-responder (expressive)	1 (2.6%)

Responder = 5–6 positive responses out of 21 IAPS images; Responder (expressive) = 5–9 positive responses and 5–10 false positives; Non-responder = 0–3 positive responses out of 21 IAPS images; Non-responder (expressive) = 4 positive responses and 8 false positives

### Association between self-rated emotion and IAPS target emotions

**Fig. 5:**
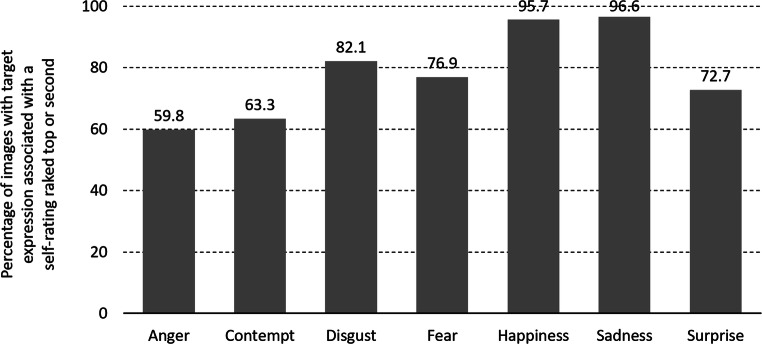
Comparison of emotion self-rating vs. IAPS category for each image used

Descriptive analysis of the self-rated emotion associated with each image provided by the participants showed that overall, 640/819 (78.1%) of IAPS images were self-rated with the target emotion ranked top or second, with rates for emotion-specific images of 59.8%, 63.3%, 82.1%, 76.9%, 95.7%, 96.6% and 72.7% for anger, contempt, disgust, fear, happiness, sadness and surprise respectively (Fig. 5).

## Conclusions and discussion

Good correspondence of the IAPS-designated target emotion with the self-ratings of participants provides some confidence that the images used to target emotional responses in these participants were appropriate despite known concerns regarding self-report measures noted above.

Almost 30% of the images presented (236/819, 28.8%) were associated with detecting an emotional response, with almost half of these (100/819, 12.2%) associated with a positive response in line with the target expression. Positive responses were more common in association with images intended to evoke happiness and sadness (41% and 21.4% positive response rates respectively).

However, the majority of images resulted in no response by the participants. 583/819 (71.2%) of images were associated with neither a positive nor a false-positive response. Inspection of the video footage showed that in these cases facial expression following exposure to an image did not change throughout the subsequent 5-s interval. A 5-s interval was considered adequate to evoke a change in facial expression as macro expressions of emotion covering large areas of the face can typically take up to 2 s to be expressed [19], and another study exploring emotion elicitation using video and photographic stimuli used a similar time interval (6 s) in which to study response [20]. We believe the non-response rate observed to be a limitation of the experiment as opposed to a failure to support the concept of extraction of expression artefacts from video diary footage. In our experiment, we have assumed that the emotions evoked by viewing an image may be externalised by a change in facial expression. One reason for low emotion-expression coherence may be insufficient intensity of the underlying emotion to evoke an external response [21]. In addition, some facial expressions of emotions are more prone to expression in social as opposed to solitary contexts [21]. In our experiment there may be a “white coat effect” resulting in participants being less relaxed in the experimental conditions as they might be in a normal conversational situation, meaning they are less able to represent natural expressions and changes in expression in response to image presentation. In addition, participants may exhibit an element of immunity to the impact of screen-based images due to the likely amount of time participants spend being exposed to a variety of images through television and social media.

Despite this, the objective of this proof of concept study was to examine the ability of the VDMA solution to measure changes in facial expression, where they exist. This proof-of-concept study provides early encouraging findings that facial expressions derived from video footage may provide appropriate measures of expression. The non-response rate is likely a limitation of the experiment, and expression detection may be more sensitive in conversational settings and when looking at within-individual changes over time. The VDMA is expected to operate as a video diary in which patients are requested to record how they feel in their own words longitudinally before and during a treatment intervention. In depression, for example, patients may describe in their own words through short videos how they are feeling emotionally and physically, and how these feelings impact their daily life. The non-verbal aspects of this communication may be as informative as the words used themselves, and patients or a caring physician reviewing these video records may pick up on these cues in addition to the informational content delivered to aid their interpretation of patient health status. The MERET instrument, for example, has demonstrated utility in enhancing patients’ overall impression of change in health status through self-review of audio recorded self-assessments made before treatment intervention in patients with major depressive disorder [22]. As a next step we intend to evaluate the sensitivity of facial expression detection in the context of a conversational video diary in patients suffering from depression. When expression analysis is combined with voice and sentiment analysis, this may lead to novel automated measures of health status in patients using a video diary in indications including depression, anxiety, schizophrenia, autism spectrum disorder, PTSD and others.

The utility of this approach may provide valuable objective endpoints to contribute to the measurement of intervention effects in pharmaceutical clinical trials. It may also enable objective measures that can be presented alongside a video diary review interface to enable enhanced assessment and monitoring of patients by physicians during routine care for various mental health conditions.

## Electronic Supplementary Material

ESM 1 (DOCX 21 kb)

## Data Availability

Not available.
