# Supplementary material for: Proof-of-Concept Study: a Mobile Application to Derive Clinical Outcome Measures from Expression and Speech for Mental Health Status Evaluation
Source: J Med Syst. 2020 Nov 11;44(12):209. doi: 10.1007/s10916-020-01671-x (PMC7658062; doi:10.1007/s10916-020-01671-x)
Supplement: Supplementary file 1 — (DOCX 21 kb) [file 10916_2020_1671_MOESM1_ESM.docx]

**Online Resource 1**

**Listing of Images from the International Affective Picture System (IAPS) database * used in the proof-of-concept study**

| **Emotion Category** | **IAPS Image ID Number** | **Basic Image Descriptor** |
| --- | --- | --- |
| Surprise | 4690 | Erotic Couple |
|  | 4693 | Erotic Couple |
|  | 8300 | Pilot |
| Happy | 2045 | Baby |
|  | 1463 | Kittens |
|  | 1610 | Rabbit |
| Sadness | 9220 | Cemetery |
|  | 2205 | Hospital |
|  | 2301 | Kid Cry |
| Disgust | 9325 | Vomit |
|  | 3266 | Injury |
|  | 3213 | Surgery |
| Fear | 1120 | Snake |
|  | 1201 | Spider |
|  | 1300 | Pitbull |
| Contempt | 2981 | Deer Head |
|  | 9800 | Skinhead |
|  | 9810 | KKK Rally |
| Anger | 9940 | Explosion |
|  | 6313 | Attack |
|  | 6520 | Attack 2 |

* Lang P, Bradley M, Cuthbert B (2008) International affective picture system (IAPS): Affective ratings of pictures and instruction manual. Technical Report A-8, University of Florida, Gainesville, FL, USA
